# How Perceived Pain Influence Sleep and Mood More Than The Reverse: A Novel, Exploratory Study with Patients Awaiting Total Hip Arthroplasty

**DOI:** 10.3389/fpsyg.2016.01689

**Published:** 2016-10-28

**Authors:** Tone Blågestad, Ståle Pallesen, Janne Grønli, Nicole K. Y. Tang, Inger H. Nordhus

**Affiliations:** ^1^Department of Clinical Psychology, University of BergenBergen, Norway; ^2^Department of Psychosocial Science, University of BergenBergen, Norway; ^3^The Norwegian Competence Center for Sleep Disorders, Haukeland University HospitalBergen, Norway; ^4^Department of Biological and Medical Psychology, University of BergenBergen, Norway; ^5^Department of Psychology, University of WarwickCoventry, UK; ^6^Department of Behavioural Sciences in Medicine, University of OsloOslo, Norway

**Keywords:** chronic pain, sleep, mood, attribution, reciprocal relationships between symptoms

## Abstract

**Objectives:** Attributions about how comorbid symptoms worsen or improve each other are central cognitive components of chronic pain that are shown to facilitate or impede the recovery process. Still, these attributions have been poorly illuminated in chronic pain patients. The present study explored perceptions of how sleep, pain, and mood influence each other in patients awaiting total hip arthroplasty (THA).

**Design and Methods:** In this cross-sectional study, 291 patients (mean age 67.8, 65.3% female) rated 12 statements about how much a given symptom (pain, sleep, mood) changed when another symptom (pain, sleep, mood) worsened or improved on a response scale ranging from much worse (-2) via no change (0) to much better (2). Sleep (Bergen Insomnia Scale), pain (McGill Pain Questionnaire), anxiety and depression (Hospital Anxiety and Depression Scale) were assessed as background variables.

**Results:** Of the patients in the study, 56% reported symptoms indicating insomnia. Anxiety and depression were indicated in 16 and 10%, respectively. Over 80% rated their pain as horrible/unbearable and reported that pain occurred always/daily. When experiencing increased pain, a majority perceived that sleep (90%) and mood (70%) worsened, whilst experiencing reduced pain improved sleep and mood in 50%. Poor sleep increased pain and worsened mood in 45 and 60% of the patients, respectively. Better sleep was perceived to reduce pain and improve mood in 50%. Worsened mood increased pain (46%) and worsened sleep (52%). Improved mood decreased pain and improved sleep in 25 and 35%, respectively.

**Discussion:** In this study, a novel approach was used to investigate perceptions of reciprocal relationships between symptoms. We found that THA patients perceived interrelationships between pain, sleep and mood. These perceived interrelations were stronger when symptoms worsened than when symptoms improved. They also held stronger beliefs about the effect of pain on sleep and mood, than the effect of sleep and mood on pain. Attributions are central in illness perception and ultimately affect illness behavior. For patients who perceive symptoms to interrelate, the door has already been opened to utilize these attributions in treatments aiming to disrupt vicious cycles, hence supporting the use of multimodal treatments.

## Introduction

Pain in patients eligible for total hip arthroplasty (THA) is normally caused by arthritis (Hamel et al., 2008). The experience and expression of such pain is commonly modulated by the presence of comorbid conditions like sleep and mood disturbances (Chiu et al., 2005; Lautenbacher et al., 2006; Roehrs et al., 2006; Haack et al., 2007; Smith et al., 2007; O’Brien et al., 2011; Blagestad et al., 2012) as well as expectancies and appraisals about these conditions (Tracey, 2010; Bjorkedal and Flaten, 2012). Chronic pain patients often attribute specific causal relationships in terms of how these conditions influence each other (Morin et al., 1998; Hawker et al., 2008; Tang et al., 2009; Theadom and Cropley, 2010). Shown to shape symptom expression, such attributions also influence a person’s overall perceived symptom load (Petrie et al., 2007). Attributions typically enable a person to predict and influence future events, and are, accordingly, found to predict thoughts and behavior aimed at getting well, or motivation to perform preventive health behavior (Michela and Wood, 1986). In chronic pain specifically, such attributions are found to be central cognitive facilitators or impediments to the recovery process (Dean, 1986; Michela and Wood, 1986; DeGood and Kiernan, 1996; Roesch and Weiner, 2001).

Sleep and mood disturbances are frequently experienced as a consequence of pain in chronic pain patients (Brennan and Lieberman, 2009), and often interact to worsen pain (Chiu et al., 2005; Zautra et al., 2005; Vitiello et al., 2009; Ong et al., 2010; Theadom and Cropley, 2010; Sivertsen et al., 2015). Conversely, there is also recent research highlighting the amplifying effect of improvements of sleep and mood involved in the recovery from chronic pain (Zautra et al., 2005; Davies et al., 2008; Ashworth et al., 2010; Ong et al., 2010). Sleep and mood are therefore central components both in expression of illness, and as part of the multimodality treatment of chronic pain patients. There is emerging evidence that chronic pain patients with comorbid sleep problems are aware of the bidirectional relationship between the constructs (Tang et al., 2009; Ramlee et al., 2016). Hence, there is great potential in assessing and utilizing attributions to aid accurate understanding and treatment of chronic pain and its comorbid conditions.

Attributions about the perceived relationship between pain, sleep and mood have been poorly illuminated empirically. A few studies have explored the perceived effect of pain on sleep and mood and found, first, that good sleep and emotional well-being are rated as very important for chronic pain patients (Turk et al., 2008). Furthermore, many pain patients are convinced that their sleep problems result from their pain (Morin et al., 1998; Hawker et al., 2008), and consequently when they experience severe pain, it is difficult for them to sleep (Edwards et al., 2011; Tang et al., 2012a). In line with this, chronic pain patients often believe that their sleep problem will disappear when their pain is gone (Morin et al., 1998). Of the studies to date, only one has explored this reciprocal relationship from the perspective of sleep, finding that fibromyalgia patients directly associate poor sleep with feelings of pain and fatigue, in addition to reduced coping abilities (Theadom and Cropley, 2010). Knowledge of attributions about the perceived mutual influence of mood, pain and sleep is lacking in chronic pain patients. Also missing are studies exploring attributions about how improvements, and not only worsening, of symptoms, are perceived to influence other symptoms. Finally, in order to investigate whether bidirectional relationships exist in how patients attribute reciprocal symptom influence, these multidirectional attributions need to be explored within the same individuals.

To improve our understanding of attributions of symptoms in chronic pain patients, we developed an instrument to explore how patients waiting to undergo THA perceived pain, sleep and mood to influence each other. The questionnaire contained 12 statements assessing two main aspects of symptom influence: (1) how levels of pain influence sleep and mood, but also, conversely, the influence of sleep and mood on pain, and (2) the perceived effect on pain, sleep and mood both when symptoms are worse than usual and when symptoms are better than usual. Based on the responses to these statements, bidirectional relationships between pain, sleep and mood were investigated.

## Materials and Methods

### Study Design

This questionnaire-based study was part of a prospective, multi-center study that evaluated pain, sleep, anxiety, depression and symptom attribution in patients 6–0 weeks before THA. These results are reported elsewhere.

### Participants

Participants were recruited from four different orthopedic departments in hospitals across Norway (Haukeland University Hospital, Diakonhjemmet Hospital, Coastal Hospital Hagevik and Sørlandet Hospital Arendal) between May 2014 and November 2015. A total of 643 patients who entered the waiting lists for THA were invited to participate and 314 patients accepted. The response rate differed between the hospitals, with response rates of 75.2, 72.0, 58.7, and 23.2%, respectively. Due to the low response rate in the last hospital, sensitivity analyses were performed whereby results with all hospitals included were compared to results from all hospitals without the hospital with the lowest response rate. In all cases, the results did not significantly differ, with differences in effect (measured by Cohen’s *d* effect size) of less than 0.1. Hence, including data from the hospital with low response rate had negligible effects on the results. Eighteen participants were excluded from the analysis due to missing signed consent form pre-operatively, and five because their THA was canceled. Thus, the final sample consisted of 291 participants.

### Procedure

The participants were recruited consecutively from the waiting lists for THA. When sending the notice of the date for their operation, an administrative staff member at the respective hospital enclosed information about the study, provided a questionnaire consisting of several validated scales as well as an informed consent form. Patients willing to participate were asked to complete the questionnaire at home and return the questionnaire and signed consent form when arriving at the pre-operative consultation. At one hospital, the patients were asked to return the questionnaire in a prepaid return envelope. Date of surgery was extracted from the Norwegian Arthroplasty Register via the participant’s unique identifying code provided in the questionnaire. The participant’s address was provided by the respective hospitals.

The study was approved by The Regional Committee for Medical and Health Research Ethics in Western Norway (2014/63/REK Vest) and was also approved at each of the hospitals involved.

### Materials

The questionnaire contained a selection of measures that registered the participant’s name, identifying code and data on the participant’s demographics (age, sex, ethnicity, education level, employment, income, marital status, and number of children) and self-reported health. The following clinical background variables were assessed; pain intensity and frequency [from the McGill Pain Questionnaire (Melzack, 1975), in addition to reporting additional pain in the hip being replaced], sleep [Bergen Insomnia Scale (BIS; Pallesen et al., 2008)], symptoms of anxiety and depression [Hospital Anxiety and Depression Scale (Zigmond and Snaith, 1983)], and specific hip-related outcomes [Hip Osteoarthritis Outcome Scale (Nilsdotter et al., 2003)]. In addition, the participants completed a questionnaire assessing attribution of symptoms specifically designed for this study. These questionnaires are briefly described in the following paragraphs.

General pain was assessed using two verbal descriptor scales from the McGill Pain Questionnaire (Melzack, 1975), validated in Norwegian (Kim et al., 1995). The magnitude of pain was assessed by the phrase: “place a cross in the box fitting your pain,” with the response alternatives “no pain,” “weak,” “unpleasant,” “bothersome,” “terrible” or “unbearable.” The frequency of pain was assessed by the phrase: “How often do you have pain?” The response alternatives were “constantly,” “daily,” “several times a week,” “about once a week,” “several times a month,” “about once a month,” “less than once a month” and “never.” Patients were also asked whether the pain was chronic (>3 months), if they had additional pain to the hip being replaced and whether they felt that analgesics relieved their pain.

Sleep was assessed using the BIS which measures self-reported symptoms of insomnia corresponding to the criteria for insomnia in the Diagnostic and Statistical Manual of Mental Disorders-IV-TR (American Psychiatric Association, 2000). The scale includes six items that are scored on an eight-point scale indicating the number of days per week for which a specific symptom is experienced (0–7 days, total scores ranging from 0 to 42). The BIS is validated using subjective as well as polysomnographic data and is found to possess good psychometric properties (Pallesen et al., 2008). Participants were categorized as insomniacs if scoring 3 or more on at least one of items 1–4, and 3 or more on at least one of items 5 and 6. The scale provided a Cronbach’s alpha of 0.91 in the present study.

The Hospital Anxiety and Depression Scale (HADS) was used to assess the presence of anxiety and depression. The HADS contains 14 items describing non-vegetative symptoms of anxiety and depression (scoring range 0–21 for both anxiety and depression subscales) (Zigmond and Snaith, 1983). Higher scores indicate greater symptom severity. A score of 8 or higher on the HADS subscales of anxiety and depression respectively is considered a clinical cut-off. A validated Norwegian version of the HADS was used in the present study (Bjelland et al., 2002), for which the Cronbach’s alpha for each subscale was 0.86.

The Hip Osteoarthritis Outcome Scale (HOOS) evaluated hip related outcomes through 5 subscales [pain, symptoms, functioning in activities of daily living (ADL), functioning in sport and recreation, and hip-related quality of life]. Standardized response alternatives are provided on a 5-point Likert scale (0–4). Then, a normalized score from 0 to 100 is calculated for each subscale (100 indicating no symptoms, and 0 indicating extreme symptoms) (Nilsdotter et al., 2003). The Cronbach’s alpha coefficient was 0.96 in the present study.

The main outcome variable was symptom attribution. In order to assess how participants perceived symptoms of pain, sleep and mood to influence each other, a questionnaire was developed containing 12 statements about how much a given symptom (pain, sleep, mood) changed when another symptom (pain, sleep, mood) worsened or improved. Six statements explored the effect on the other two symptoms when a given symptom worsened, and six statements explored the effect on the other two symptoms when a given symptom improved. The participants were asked to provide responses on a 5-point scale (from 1 to 5) for each statement. **Table 1** presents the 12 statements together with the response alternatives.

**Table 1:** Symptom attribution questionnaire.

	Response alternatives				
	
	Much better	A bit better	No change	A bit worse	Much worse
When my pain is worse than usual, my sleep becomes…					
When my pain is worse than usual, my mood becomes…					
					
When my sleep is worse than usual, my pain becomes…					
When my sleep is worse than usual, my mood becomes…					
					
When my mood is worse than usual, my pain becomes…					
When my mood is worse than usual, my sleep becomes…					
					
When my pain is weaker than usual, my sleep becomes…					
When my pain is weaker than usual, my mood becomes…					
					
When my sleep is better than usual, my pain becomes…					
When my sleep is better than usual, my mood becomes…					
					
When my mood is better than usual, my pain becomes…					
When my mood is better than usual, my sleep becomes…					


### Data Analysis

Analyses were performed using SPSS, version 21. For the symptom attribution questionnaire, the rating scale was recoded in order to display the positive or negative properties of the perceived influence. *Much worse* was recoded to -2, *a bit worse* was recoded to -1, *as usual* was recoded to 0 (indicating no change), *a bit better* was recoded to 1 and *much better* was recoded as 2. Descriptive statistics were used to characterize symptom attributions and the difference of the mean from 0 (no change) was measured through one-sample *t*-tests. A paired sample *t*-test was used to compare items in bidirectional relationships in order to assess the directionality of symptom attribution. All statements are listed in **Table 1.** For example, whether pain influences sleep more than sleep influences pain was assessed by comparing statements 1a and 2a for the worsening relationships between symptoms, and statements 1c and 2c for the improving relationships between symptoms. For the pain-mood relationship, statements 1b and 3a and statements 1d and 3c were compared for the worsening and improving effect of symptoms, respectively. For the sleep-mood relationship, statements 2b and 3b and statements 2d and 3d were compared for the worsening and improving effect of symptoms, respectively. Pairs with one or more missing values were removed from analyses (excluded pairwise). To measure the magnitude of the effect, effect sizes (Cohen’s *d*) were estimated using DSTAT (Johnson, 1995). An effect size of 0.2 is regarded as a small, 0.5 a medium, and effect sizes of 0.8 or higher are regarded as large (Cohen, 1988). A Bonferroni-correction was applied due to multiple comparisons, setting the new critical *p*-value to 0.002.

## Results

### Description of Baseline Characteristics

**Table 2** presents the participants’ characteristics. The mean age was 67.9 years and 65.3% were female. The majority were retired, married/cohabiting, and had 2 or 3 children. The majority had an income between 100 000 and 399 999 NOK (equivalent to approximately 12 000–50 000 USD). Clinical background variables are presented in **Table 3.** On the PPI, most participants rated their pain to be horrible (66.3%) or unbearable (16.2%), and over 90% rated their pain to occur daily or be present constantly. Over 70% also reported additional pain in the hip being replaced. In total, 54.0% reported symptoms indicating insomnia (the average BIS score was 16.4, *SD* = 12.0). Symptoms indicating caseness of anxiety or depression were reported by 16.2 and 10.3%, respectively. According to the hip-specific outcome measure (HOOS), the self-reported hip-related pain, function, quality of life, ADL and sports and recreation were poor (between 40 and 24 on a scale of 100–0 where 100 indicates no symptoms, and 0 indicates extreme symptoms).

**Table 2:** Demographics (*N* = 291).

Age	67.9 (*SD*: 11.1), range 23–96
Sex	65.3% female
**Education (%)**	
No schooling completed	0.3
Nursery school	15.8
High school graduate	16.2
Trade/technical/vocational training	29.2
Bachelor’s degree	25.4
Master’s degree	10.1
Doctorate degree	1.0
**Work status (%)**	
Full time 100 %	15.1
Part time	3.4
Homemaker	0.7
Unemployed	0.7
Student	0.3
On sick leave 100%	7.6
On sick leave <100%	1.7
Work assessment allowance	1.4
Disability benefit	8.6
Retired	59.8
**Marital status (%)**	
Married/cohabitant/partner	74.6
Single/separated/divorced/widow/widower	24.7
**Number of children (%)**	
None	9.9
1–2	48.1
3–4	36.4
5 or more	5.2
**Income (NOK, %)**	
0–199 999	15.4
200 000–399 999	44.7
400 000–599 999	25.1
600 000 or more	8.6


**Table 3:** Clinical background variables (*N* = 291).

Pain	
**Magnitude (%)**	
Weak, unpleasant or bothersome	14.5
Horrible	66.3
Unbearable	16.2
**Frequency (%)**	
Constant or daily	89.3
Once or multiple times a week	7.2
Once or multiple times a month	0.6
Less than once a month	1.0
Chronic (pain lasting <3 months, %)	96.9
Experiencing additional pain to the replaced hip (%)	70.4
**Effect of analgesics (%)**	
None or to a small degree	56.0
To a large degree or completely	34.7
**Health (%)**	
Excellent or very good	24.0
Good or quite good	64.3
Poor	10.7
**Health compared to a year ago (%)**	
Much or a bit better than a year ago	7.2
About the same as a year ago	31.3
A bit or much worse than a year ago	59.1
**Insomnia (BIS)**	
Sum (mean)	16.4
Cutoff-insomnia^∗^ (%)	54.0
Anxiety (HAD–A < 8, %)	16.2
Mean (*SD*)	4.3 (*SD*: 3.9)
Depression (HAD–D < 8, %)	10.3
Mean (*SD*)	3.5 *(SD*: 3.19)
**Hip related measures - HOOS**	
Symptoms	34.35
Pain	39.22
ADL	40.05
Sportrec	23.02
QoL	24.78


*^∗^As defined by DSM-IV; BIS, Bergen Insomnia Scale; HADS, Hospital Anxiety and Depression Scale; HOOS, Hip Osteoarthritis Outcome Scale. Normal scores from 0–100, (100 indicating no symptoms, and 0 indicating extreme symptoms).*

### Attributions between Pain, Sleep and Mood When Symptoms Worsened

**FIGURE 1:**
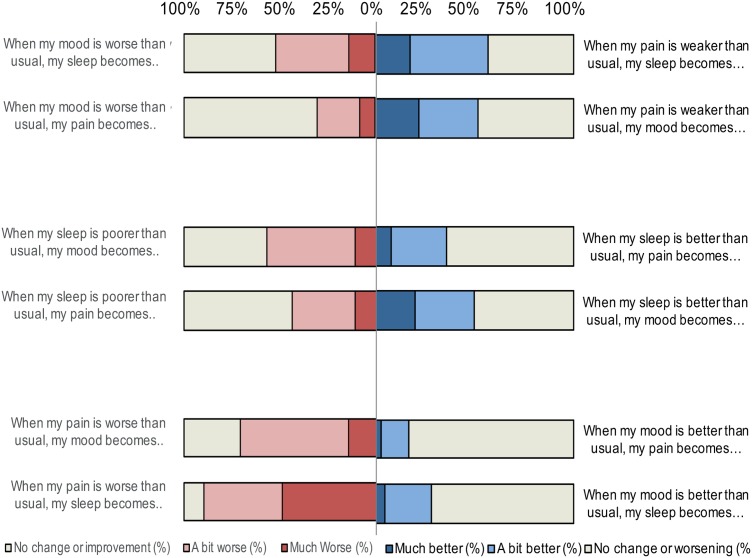
**Percentages of patients attributing changes in their pain, sleep, and mood when symptoms worsen or improve**.

A substantial portion of patients perceived that worsening of symptoms influenced their pain, sleep and mood (**Table 4**, **Figure 1**). Ninety per cent of the patients reported that sleep worsened in the presence of increased pain and 70% reported mood to worsen with increased pain. Close to 45% perceived their pain to worsen with poorer sleep, and almost 60% perceived mood to worsen with poorer sleep. Worse mood was perceived to have the least influence on pain (64.3% perceived there to be no change), but 51.9% reported mood to influence sleep. As displayed in **Table 5**, the mean on all subscales differed significantly from 0 (all *t*-values significant on the 0.002-level) with effect sizes ranging from 0.6 to 2.3 (medium to very large effect size).

**Table 4:** Description of attributions of the effect between pain, sleep and mood (*N* = 291).

Attributions when symptoms worsen			Level of effect (%)				
			
	Mean (from -2 to 2)	*SD*	Much worse	A bit worse	No change	A bit better	Much better
When my pain is worse than usual, my sleep becomes…	-1.4	0.6	49.1	40.9	6.5	0.0	0.0
When my pain is worse than usual, my mood becomes…	-0.9	0.6	14.4	56.4	24.1	0.3	0.0
When my sleep is poorer than usual, my pain becomes…	-0.6	0.7	11.3	32.6	51.9	0.3	0.0
When my sleep is poorer than usual, my mood becomes…	-0.7	0.7	11.3	45.7	37.5	0.3	0.0
When my mood is worse than usual, my pain becomes…	-0.4	0.6	7.9	22.0	64.3	0.0	0.0
When my mood is worse than usual, my sleep becomes…	-0.7	0.7	13.4	38.5	41.2	0.3	0.0

**Attributions when symptoms improve**			**Level of effect (%)**				
			
	** Mean (from -2 to 2)**	***SD***	**Much better**	** A bit better**	** No change**	**A bit worse**	**Much Worse**

When my pain is weaker than usual, my sleep becomes…	0.7	0.9	17.5	39.2	30.6	6.5	0.3
When my pain is weaker than usual, my mood becomes…	0.8	0.8	21.6	30.2	40.5	1.4	0.3
When my sleep is better than usual, my pain becomes…	0.4	0.7	7.6	27.8	56.4	2.7	1.4
When my sleep is better than usual, my mood becomes…	0.7	0.8	18.9	28.2	46.4	1.0	0.3
When my mood is better than usual, my pain becomes…	0.2	0.5	2.7	13.7	74.9	2.1	0.7
When my mood is better than usual, my sleep becomes…	0.3	0.6	4.5	23.4	63.2	3.1	0.0


**Table 5:** Strength of relationships between symptoms

Attributions when symptoms worsen	Difference from 0 (indicating no change)					
	
	*t*	*df*	95% CI of the difference		Sig	Effect size
When my pain is worse than usual, my sleep becomes…	-39.0	280	-1.5	-1.4	0.000	2.3
When my pain is worse than usual, my mood becomes…	-23.2	276	-1.0	-0.8	0.000	1.4
When my sleep is poorer than usual, my pain becomes…	-13.7	279	-0.7	-0.5	0.000	0.8
When my sleep is poorer than usual, my mood becomes…	-17.8	275	-0.8	-0.6	0.000	1.1
When my mood is worse than usual, my pain becomes…	-10.4	273	-0.5	-0.3	0.000	0.6
When my mood is worse than usual, my sleep becomes…	-16.1	271	-0.8	-0.6	0.000	1.0

**Attributions when symptoms improve**	**Difference from 0 (indicating no change)**					
	
	***t***	***df***	**95% CI of the difference**		**Sig**	**Effect size**

When my pain is weaker than usual, my sleep becomes…	13.7	273	0.6	0.8	0.000	0.8
When my pain is weaker than usual, my mood becomes…	15.1	273	0.7	0.9	0.000	0.9
When my sleep is better than usual, my pain becomes…	8.9	278	0.3	0.5	0.000	0.5
When my sleep is better than usual, my mood becomes…	13.8	275	0.6	0.8	0.000	0.8
When my mood is better than usual, my pain becomes…	5.2	273	0.1	0.2	0.000	0.3
When my mood is better than usual, my sleep becomes…	8.4	273	0.2	0.4	0.000	0.5


*CI, Confidence Interval. Effect size, Cohen’s d.*

### Attributions between Pain, Sleep and Mood When Symptoms Improved

Patients reported improvement of one symptom to influence the other symptoms to a smaller degree than did worsening of it (**Table 4**, **Figure 1**). Still, reduced pain was perceived to improve sleep and mood in 56.7 and 51.8% of the patients, respectively. Improved sleep was also perceived to improve pain in 35.4% of the patients. Improved sleep had a strong influence on improvements of mood and was reported by 47.2% of the patients. Again, mood was perceived to have the least influence on pain and sleep; improved mood was perceived not to have an effect in 74.9% for pain and 63.2% for sleep. Regardless, all variables differed significantly from 0 (all *t*-values significant on a 0.002-level). **Table 5** displays the effect sizes (ranging from 0.3 = small effect size to 0.9 = large effect size).

### Directionality of Attributions When Symptoms Worsened

**FIGURE 2:**
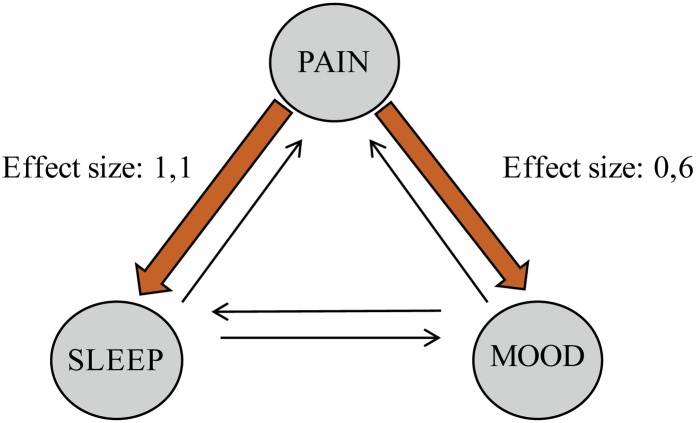
**Perceptions about reciprocal relationships when symptoms worsen**.

When symptoms worsened, pain was significantly perceived to influence sleep more than sleep influenced pain (*t* = -19.2, *df* = 279). The effect size was large (*d* = 1.1) (**Table 6**, **Figure 2**). Increased pain was also perceived to influence mood significantly more than worsened mood influenced pain (*t* = -10.5, *df* = 269). This effect size was medium (*d* = 0.6). There was no significant difference to which degree the participants perceived sleep and mood to influence each other.

**Table 6:** Directionality of symptom attribution between pain, sleep and mood (*N* = 291).

When symptoms worsen		95 % CI		*t*	df	Sig	Effect size
Pain affects sleep (1a)		-1.0	-0.8	-19.2	279	0.000	1.1
Sleep affects pain (2a)							

Pain affects mood (1b)		-0.6	-0.4	-10.5	269	0.000	0.6
Mood affects pain (3a)							

Sleep affects mood (2b)		-0.1	0.1	-0.17	267	0.868	
Mood affects sleep (3b)							

**When symptoms Improve**		**95 % CI**		****t****	***df***	**Sig**	**Effect size**

Pain affects sleep (1c)		0.2	0.4	5.7	272	0.000	0.4
Sleep affects pain (2c)							

Pain affects mood (1d)		0.5	0.7	10.3	268	0.000	0.6
Mood affects pain (3c)							

Sleep affects mood (2d)		0.3	0.5	8.0	269	0.000	0.5
Mood affects sleep (3d)							


*CI, Confidence Interval. Effect size, Cohen’s *d*.*

### Directionality Attributions When Symptoms Improved

**FIGURE 3:**
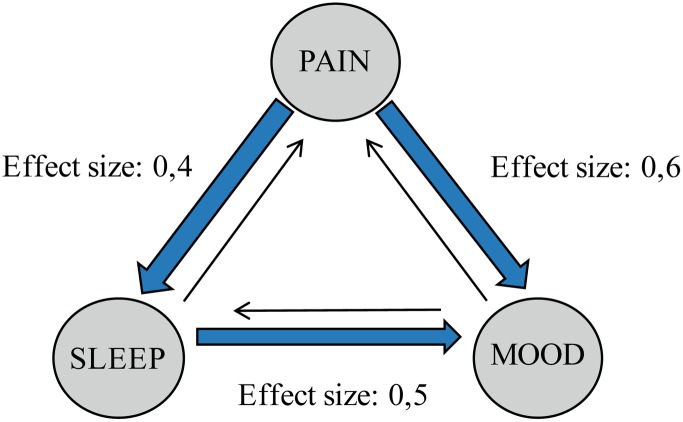
**Perceptions about reciprocal relationships when symptoms improve**.

Reduced pain significantly influenced sleep more than improved sleep influenced pain (**Table 6**, **Figure 3**, *t* = 5.7, *df* = 272). The effect size was small to medium (*d* = 0.4). Reduced pain also influenced mood more than improved mood influenced pain (*t* = 10.3, *df* = 268) with a medium effect size (*d* = 0.6). Lastly, improved sleep was perceived to influence mood more than improved mood influenced sleep (*t* = 8.0, *df* = 269) with a medium effect size (*d* = 0.5).

## Discussion

In contrast to the number of studies that aim to disentangle the relationship between chronic pain, sleep and mood, limited effort has been devoted to investigating how patients themselves perceive how these symptoms influence each other. The present study explored perceived bi-directional influences of pain, sleep and mood when symptoms worsened or improved in patients awaiting THA. We found that a large majority perceived sleep and mood to worsen when experiencing worse pain than usual and less intense pain than usual was perceived to improve sleep and mood. A significant proportion of the patients perceived pain to worsen with poorer sleep, and better sleep was perceived to reduce pain. Overall, pain stood out as the symptom with the largest perceived influence on the other symptoms, while mood was the symptom perceived by the fewest patients as influencing the other symptoms.

### Worsening Symptom Attribution

We found that almost all of the patients in the present study perceived increased pain to lead to poorer sleep, corroborating the impact of pain on sleep in previous qualitative and quantitative studies (Smith et al., 2000; Breivik et al., 2006; Hawker et al., 2008; Ashworth et al., 2010; Theadom and Cropley, 2010; Henderson et al., 2013; Thomazeau et al., 2014). For example, many chronic pain patients firmly believe that when they are in pain, it is simply impossible for them to get comfortable and go to sleep (Edwards et al., 2011; Tang et al., 2012a). The rate of patients perceiving pain to negatively impact sleep was higher in the present study than found in chronic pain patients in general (90% vs. 65%) (Breivik et al., 2006); also, the intensity and frequency of pain was higher in the present sample. The present study highlights the importance of effective treatments for chronic pain.

One third of our patient’s perceived pain and mood to worsen with poorer sleep, mirroring one qualitative study where a poor night’s sleep was found to be directly associated with increased pain (Theadom and Cropley, 2010). Our results are also in line with increasing numbers of observational and experimental studies establishing an effect of sleep on pain. However, more than half of the patients in our study did not perceive poorer sleep to increase pain. One of the most distressing features of chronic pain is the unpredictable fluctuation in its type and intensity (Hawker et al., 2008), and it may thus be difficult for patients to perceive how these symptoms are influenced by sleep and mood. This is supported by a recent daily process study that reported the pain-relieving effect of good sleep to be short-lived. Although sleep quality showed an inverse relationship with pain upon waking and during the first half of the day, no association was found during the second half of the day (Tang et al., 2012c). The authors suggest that for some patients, reduced pain might actually lead to over-extending activity. This would cause even more pain during the night, consequently masking the positive effect of good sleep on pain. Hence, perceived improvement of pain as a result of good sleep might be masked by the fluctuations or other sources of increasing pain. In addition, many clinicians do not regularly assess, diagnose or treat comorbid sleep problems in pain patients, since they are under the false impression that treatment of the underlying organic/psychiatric condition will resolve any residual sleep complaints (Ozminkowski et al., 2007). This lack of focus might contribute to these patients’ perception of illness.

Although depression, anxiety and negative mood are closely related to chronic pain (Lin et al., 2003; Argoff, 2007; Montin et al., 2007; O’Brien et al., 2010; Wylde et al., 2011; Hoogeboom et al., 2012), worse mood than usual was perceived by the fewest patients to impact pain and sleep in our study. In a study of middle-aged women with chronic pain, an increase in negative affect during the previous week predicted greater pain during subsequent weeks (Zautra et al., 2005). One could assume that patients would perceive this same effect to a larger degree than what we found. Our results might indicate, as suggested by Lavigne (2005), that negative mood affects sleep and pain in a more indirect way. Alternatively, if the perception about reciprocal relationships between symptoms depend on the presence of the symptom in question, our results might simply reflect lower rates of anxiety and depression compared to pain and sleep complaints in the present study. Future investigations of symptom attributions in chronic pain patients with larger samples of comorbid anxiety and depression would clarify this matter.

### Improving Symptom Attribution

The present study is to the authors’ knowledge the first to explore how improvement in one symptom (pain, sleep, or mood) is perceived to influence other symptoms. We found that a majority of our patients perceive reduction of pain to improve sleep and mood. Although chronic pain is intractable by definition, this underlines the importance of optimal pain management, whereby reducing pain may also improve comorbid symptoms (Turk and Cohen, 2010). More noteworthy is the finding that one third perceived better sleep than usual to improve pain. The role of sound sleep is key in chronic pain patients. Firstly, restorative sleep is shown to be involved in the resolution of chronic pain (Davies et al., 2008), and chronic pain patients that are “good sleepers” report less pain at night, less negative consequences from their pain and less depression or pain-related anxiety (Ashworth et al., 2010). Accordingly, the concurrent treatment of pain-related sleep problems is found either to reduce pain itself, or to reduce pain interference, which might be an important aspect of pain in chronic pain patients (Edinger et al., 2005; Vitiello et al., 2009; Jungquist et al., 2010; Tang et al., 2012b). Furthermore, positive emotions are seen as resilience factors decreasing the negative impact of chronic pain conditions (Zautra et al., 2005; Ong et al., 2010). In a study investigating positive and negative affect in women with chronic pain, people who tend to have higher levels of positive affect also had less pain over time (Zautra et al., 2005). Hence, adequate sleep and positive mood seems to be a buffer involved not only in the biological foundation of pain perception (Davies et al., 2008), but also in the ability to cope with daily pain (Theadom and Cropley, 2010). Positive emotions and good sleep may therefore play an important role in fostering recovery after episodes of severe pain (Zautra et al., 2001).

Taken together, the present findings have implications for the assessment and treatment of chronic pain and pain-related sleep and mood disturbances. That symptoms interact to worsen and improve each other forms the basis of multimodality treatments. This emphasizes the benefit of interventions aiming at disrupting vicious circles between symptoms (Argoff, 2007; Smith et al., 2009). The results of the present study support the use of interventions that target sleep and mood in addition to pain. Furthermore, attributions are found to be central cognitive facilitators or impediments to the recovery process (Dean, 1986; DeGood and Kiernan, 1996; Roesch and Weiner, 2001). According to attribution theory, individuals with chronic illness who make internal, unstable and controllable attributions also believe they can do something to minimize the impact of their illness. This leads directly to certain motivated coping cognitions and behavior, and ultimately to more positive psychological adjustment (Weiner, 1985). For chronic pain patients who perceive symptoms to interrelate, the door has already been opened to utilize these attributions in the treatment of chronic pain and its comorbid conditions. For our patients awaiting THA specifically, these attributions might aid a positive reinforcing cycle of symptom improvement when pain is reduced after surgery.

The limitations of the study should be noted. Firstly, due to the lack of previous studies that include the key attribution elements aimed at in the present study, a questionnaire was constructed for this purpose. It is therefore not previously validated. The questions used for assessing reciprocal relationships between pain, sleep and mood should be validated in other types of samples (e.g., normal subjects as well as in patients suffering from sleep and mood disorders). In the process of developing the questionnaire, mood was intentionally chosen as a general symptom-effector instead of specifying anxiety and depression, for several reasons. By broadening the term into “mood,” we are convinced that aspects of disturbed mood such as “helplessness” or “frustrations” often experienced by these patients would be included in addition to aspects of anxiety and depression. Furthermore, there is no equivalent positive category to diagnoses such as anxiety and depression, and we also wanted to capture eventual positive attributions of improved mood, beyond the absence of negative symptoms. Another limitation is that since the patients completed the questionnaires without assistance from the researchers we had no way to ensure that participants understood the intention of the attribution questionnaire. Third, it is important to note that one of the hospitals included in the study had a very low response rate (22%), due to unknown factors. In order to ensure representativeness of our data, sensitivity analyses were performed and showed no major changes in results when the respective hospital was removed from analyses.

Despite the limitations, there are several strengths of this novel study. It places itself in a line of studies focusing on obtaining wider knowledge about the sleep-pain domain from the patient’s perspective (Hawker et al., 2008; Turk et al., 2008), but it extends the scope to also explore attributions about sleep and mood, and to illuminate both the attributions related to worsening as well as improvement of symptoms. The natural path forward is to extend this newly acquired perspective into different chronic pain populations or populations where pain is a frequently experienced comorbid symptom.

## Conclusion

The present study found that patients awaiting THA perceive pain, sleep and mood to influence each other when symptoms worsen or improve. Pain was perceived to have a stronger influence on sleep and mood, than sleep and mood had on pain. Attributions of symptom dynamics as investigated in the present study may play a key role in overall pain experience and illness behavior.

## Author Contributions

TB designed the study, developed the main questionnaire, recruited the hospitals participating in the study and collected the data. She also analyzed the data and wrote the manuscript. SP supervised the project, including participating in the design of the study and developing the main questionnaire. He also took part in deciding the choice of analyses, and critically reviewed the manuscript. JG also supervised the project, including participating in the design of the study and developing the main questionnaire. she also took part in deciding the choice of analyses, and critically reviewed the manuscript. NT took part in deciding the choice of analyses, the interpretation of the results and critically reviewed the manuscript. IN was the main supervisor of the project and participated in the design of the study and the development of the main questionnaire. She also took part in deciding the choice of analyses, and critically reviewed the manuscript. All authors have approved of the final version of the manuscript to be published.

## Conflict of Interest Statement

The authors declare that the research was conducted in the absence of any commercial or financial relationships that could be construed as a potential conflict of interest.
